# Bedside screening of carbapenem-resistant *Enterobacteriaceae* using selective agar plates: A low-cost, laboratory-independent approach for rural healthcare facilities

**DOI:** 10.1371/journal.pgph.0007479

**Published:** 2026-10-15

**Authors:** The Anh Ngo, Hieu Thi Nguyen, Nhung Ngoc Hoang, Thanh Duc Nguyen, Thanh Chi Tran

**Affiliations:** 1 Department of Infectious Diseases, Viet Tiep Hospital, Hai Phong, Viet Nam; 2 HUTECH University, Ho Chi Minh city, Vietnam; 3 Hanoi University of Public Health, Vietnam; 4 University of Medicine and Pharmacy, Ho Chi Minh city, Vietnam; Prince of Songkla University, THAILAND

## Abstract

The global surge of carbapenem-resistant Enterobacteriaceae (CRE) poses a critical threat to patient safety in intensive care units (ICUs). In low- and middle-income countries, routine CRE surveillance is often limited by the lack of microbiology laboratory infrastructure and trained personnel in peripheral healthcare facilities, resulting in reliance on empirical broad-spectrum antibiotic therapy and limited capacity to detect silent transmission. This study evaluated the operational feasibility, safety, and phenotypic agreement of a decentralized, laboratory-independent bedside CRE screening protocol using pre-formulated selective chromogenic agar and a low-cost portable incubator. Dual rectal swabs were collected from 220 ICU patients at Viet Tiep Hospital, Vietnam. One sample was processed using standard centralized laboratory procedures as the reference method, while the parallel sample was processed at the bedside by frontline nursing staff, including direct plate inoculation and point-of-care incubation. Diagnostic agreement, inter-reader reliability, procedural contamination, and operational feasibility were assessed. Gastrointestinal CRE carriage was identified in 26 of 220 patients (11.8%), with the KESC group accounting for 76.9% of positive cases. The bedside protocol demonstrated 100% phenotypic concordance with standard laboratory cultures, with 100% sensitivity, specificity, positive predictive value, and negative predictive value. Blinded assessment showed complete inter-reader agreement (κ = 1.0). No environmental cross-contamination was detected on CRE-negative plates (0.0%; 95% CI: 0–4.1%). The decentralized bedside screening model, costing less than US$1 per test, demonstrated high diagnostic agreement and operational feasibility when performed by frontline nursing staff in a non-sterile ICU environment. This approach may provide an affordable alternative for CRE surveillance in resource-limited healthcare facilities where centralized microbiology laboratory infrastructure is unavailable.

## 1. Introduction

The global spread of Carbapenem-Resistant *Enterobacteriaceae* (CRE) has emerged as one of the most critical threats to modern medicine, severely compromising patient outcomes in intensive care units (ICUs) worldwide [1]. Because CRE strains are frequently resistant to nearly all available beta-lactam antibiotics, infections caused by these pathogens are associated with prolonged hospital stays, high healthcare costs, and crude mortality rates exceeding 40% to 50% [2]. The gastrointestinal tract serves as the primary reservoir for CRE, where asymptomatic colonization often precedes clinical infection and drives silent, horizontal transmission between patients via the hands of healthcare workers or contaminated environmental surfaces [3].

To curb the spread of healthcare-associated CRE outbreaks, international guidelines strongly advocate for active surveillance programs, particularly through routine rectal swab screening of high-risk patients upon ICU admission (Hardy [4]. Early identification of gastrointestinal carriage allows for the immediate implementation of contact precautions, patient cohorting, and stricter antimicrobial stewardship. Traditionally, this screening relies on selective culture media or molecular assays processed within centralized microbiology laboratories. While clinical risk-scoring systems can help stratify patients, they lack definitive microbiological confirmation, and molecular platforms like polymerase chain reaction (PCR) or gold nanoparticle-based rapid tests remain cost-prohibitive for large-scale, routine screening in resource-constrained environments [5].

Crucially, the primary bottleneck in managing CRE within low- and middle-income countries (LMICs) is not merely financial, but structural. In many rural, district, and primary healthcare facilities across LMICs, functional microbiology laboratories and trained laboratory personnel are completely absent [6]. Consequently, screening for multidrug-resistant organisms is virtually non-existent at the peripheral levels of healthcare delivery. When critically ill patients present to these rural ICUs, clinicians are forced to rely entirely on untargeted, empirical broad-spectrum antibiotic therapies [7]. This reliance creates a vicious cycle: unguided antibiotic pressure accelerates the selection of resistant strains, while the lack of local diagnostic data leaves healthcare facilities blind to emerging outbreaks within their wards.

To bridge this critical infrastructure gap, there is an urgent need for decentralized, laboratory-independent diagnostic workflows that can shift screening capabilities directly to the point of care. In our previous work, we successfully formulated and validated a selective chromogenic agar optimized for CRE detection, demonstrating a baseline sensitivity of 97.4% and a specificity of 98.8% against molecularly characterized reference strains [8]. Furthermore, the structural feasibility and basic thermal stability of a localized, low-cost portable incubation setup designed to bypass standard laboratory requirements have been established in regional settings [9].

Building upon these foundational steps, this study evaluates the feasibility and clinical utility – operational feasibility and diagnostic agreement - of an entirely decentralized, bedside CRE culture screening protocol costing less than 1 USD per test. Within this framework, operational feasibility is defined as the capability of frontline nursing staff to execute direct sample inoculation and manage localized incubation workflows within active, non-sterile ward environments without institutional cross-contamination. Diagnostic agreement refers to the phenotypic concordance (sensitivity, specificity, and predictive values) of the bedside protocol benchmarked against standard centralized laboratory cultures. By transferring these technical tasks directly to frontline clinical personnel within a proof-of-concept hospital ICU framework, we demonstrate a practical, economical, and robust screening strategy designed to empower resource-limited healthcare facilities to implement timely infection control measures without relying on centralized laboratory infrastructure.

## 2. Methods

### 2.1. Ethics statement

The study protocol was approved by the Review Boards of Viet-Tiep Hospital, Hai Phong city (Ref No: 09/GCN-HĐĐĐ). Informed consent was obtained from all participating patients or their legal surrogates prior to sample collection.

### 2.2. Study design and clinical settings

This cross-sectional comparative study enrolled a consecutive cohort of all critically ill ICU patients admitted to Viet-Tiep Hospital from 10 October 2025 (start date) to 10 May 2026 (end date) who met screening criteria (prolonged hospitalization, prior broad-spectrum antibiotic exposure, or mechanical ventilation). Total consecutive sampling (*N* = 220) was chosen to reflect real-world clinical ICU admissions. A post-hoc precision calculation confirms that *N =* 220 yields a margin of error of ± 4.25% for the observed 11.8% CRE carriage rate at a 95% *CI*.

### 2.3. Bedside screening protocol (task-shifting model)

To evaluate a model independent of traditional infrastructure, the primary inoculation and early incubation steps were shifted entirely to frontline clinical nursing staff within the active, non-sterile ICU ward environments. Prior to study commencement, nurses underwent a brief, standardized 30-minute operational training session covering aseptic swab handling, selective plate streaking techniques, and visual color-differentiation criteria.

For each patient, a bedside rectal swab was collected using a sterile cotton swab and immediately inoculated directly onto a pre-poured, room-temperature selective chromogenic agar plate at the point of care. The inoculated plates were immediately transferred to a localized, low-cost portable incubator kept within the clinical unit.

### 2.4. Incubation framework and validation baseline

Rather than utilizing traditional laboratory infrastructure, the bedside culture plates were maintained at 37 °C for 18–24 hours inside a specialized portable incubation device. The basic structural assembly, thermal response stability, and field safety profile of this localized incubation setup under ambient tropical environments were previously established and validated [9]. The selective chromogenic medium utilized was formulated and optimized to target carbapenemase-producing strains based on specific enzymatic cleavage, a formulation previously validated against molecularly characterized reference strains containing confirmed resistance markers (such as bla_KPC and bla_NDM), yielding an established baseline screening sensitivity of 97.4% and specificity of 98.8% [8].

### 2.5. Reference laboratory protocol (control standard)

To establish a definitive scientific control comparison, a parallel, duplicate rectal swab was collected from each patient during the same clinical examination session. This control sample was placed into an appropriate transport medium and transferred immediately via standard logistics to the centralized, accredited institutional microbiology laboratory.

Upon arrival, the samples were processed using standard laboratory protocols. The control swabs were inoculated onto standard selective plates and incubated at 37 °C under controlled aerobic conditions for 18–24 hours using a compliant laboratory forced-convection incubator (Model IFA-170–8, Esco Lifesciences, Indonesia) satisfying DIN 12880 Class 3.1 thermal safety parameters.

### 2.6. Phenotypic identification and comparative analysis

Following the incubation window, both the bedside-incubated plates and the reference laboratory control plates were evaluated blindly by independent observers. Phenotypic positive identification for target CRE was determined strictly via colony color morphology on the selective chromogenic agar matrix:

- **Deep pink/red colonies** were classified presumptive for *Escherichia coli*.
- **Metallic blue/indigo colonies** were classified presumptive for the *Klebsiella, Enterobacter, Serratia, and Citrobacter* (KESC) group.

The diagnostic performance of the decentralized bedside screening protocol—including sensitivity, specificity, positive predictive value (*PPV*), and negative predictive value (*NPV*)—was calculated using the centralized reference laboratory culture results as the gold standard. Furthermore, cross-contamination rates under non-sterile clinical ward environments were monitored by checking for any non-target contaminant bacterial growth or fungal overgrowth on negative culture matrices.

### 2.7. Reader calibration and inter-observer agreement

Visual interpretation of the selective chromogenic agar matrix relies on distinct chromatic colony differentiation. Prior to study initiation, participating ICU nurses and laboratory technicians completed a standardized training session. During the trial, all bedside and reference plates were independently evaluated by two blinded assessors (a trained ward nurse and a senior laboratory technician). Inter-reader agreement was 100% (kappa = 1.0), confirming that visual interpretation of this selective medium is highly reproducible and requires minimal specialized training.

## 3. Results

### 3.1. Cohort characteristics and clinical demographics

A total of 220 critically ill patients admitted to the ICUs were enrolled in this prospective validation study. The baseline clinical demographics and risk factors for CRE colonization among the participating patients are summarized in Table 1. Prolonged prior exposure to broad-spectrum antibiotics (specifically carbapenems and third-generation cephalosporins) was heavily prevalent across both sites, matching the classic profile of high-risk ICU populations.

**Table 1:** Baseline demographic data, risk factors, and gastrointestinal Carbapenem-Resistant Enterobacteriaceae (CRE) carriage rates among critically ill ICU patients enrolled in the study (*N* = 220). Gastrointestinal carriage was confirmed via parallel rectal swab screening (See S1 Data for details).

Clinical Parameter	Value
Age (Years), Mean ± *SD*	62.4 ± 14.2
Gender, *n* (%)	
Male	111 (50.4%)
Female	109 (49.6%)
Prior Mechanical Ventilation, *n* (%)	162 (73.6%)
Prior Antibiotic Exposure (>48h), *n* (%)	199 (90.4%)
CRE Positive Phenotype, *n* (%)	26 (11.8%)
KESC Group (Metallic Blue)	20 (76.9%)
*Escherichia coli* (Deep Pink)	6 (23.1%)

*Abbreviations:* ***CRE****: Carbapenem-Resistant Enterobacteriaceae;* ***KESC group****: Klebsiella, Enterobacter, Serratia, and Citrobacter;* ***ICU****: Intensive Care Unit;* ***SD****: Standard Deviation;* ***n****: Sub-sample count;* ***N****: Total cohort size.*

*Note: Continuous variables are presented as Mean ± SD, and categorical variables are presented as counts and percentages (n, %). At N = 220, the observed CRE carriage rate of 11.8% carries a 95% confidence interval of 7.85% to 16.86% (margin of error = ±4.25%).

### 3.2. Clinical utility: diagnostic accuracy and performance metrics

Of the 220 patients screened via the parallel protocols, a significant burden of gastrointestinal CRE carriage was detected with prevalence rate of 11.8% (26/220). Among the positive cultures, taxonomic distribution based on colony color morphology on the selective chromogenic agar matrix revealed an overwhelming predominance of the *Klebsiella, Enterobacter, Serratia, and Citrobacter* (KESC) group, which accounted for 76.9% of the total positive isolates (*n =* 26). The remaining 23.1% of the positive cases (*n =* 6) exhibited deep pink/red colony phenotypes indicative of *Escherichia coli*.

To evaluate the clinical utility of the laboratory-independent screening approach, the diagnostic performance of the task-shifted bedside culture was directly benchmarked against the gold-standard centralized reference laboratory. The decentralized protocol demonstrated absolute phenotypic alignment (100% concordance) across all validated samples. As detailed in Table 2, the bedside protocol achieved a diagnostic sensitivity, specificity, *PPV*, and *NPV* of exactly 100.0%.

**Table 2:** Diagnostic performance metrics and procedural safety parameters of the laboratory-independent bedside CRE screening protocol executed by frontline nursing staff compared to the standard centralized microbiology laboratory culture.

Diagnostic Metric	Value	95% Confidence Interval (*CI*)
Sensitivity	100.0%	(90.7% – 100.0%)
Specificity	100.0%	(95.8% – 100.0%)
Positive Predictive Value (*PPV*)	100.0%	(90.7% – 100.0%)
Negative Predictive Value (*NPV*)	100.0%	(95.8% – 100.0%)
Environmental Contamination Rate	0.0%	(0.0% – 4.1%)
Procedural Execution Personnel	Frontline Nurses	Task-shifted within open ICU wards

*Abbreviations:* **CI**: Confidence Interval; **PPV**: Positive Predictive Value; **NPV**: Negative Predictive Value; **CRE**: Carbapenem-Resistant Enterobacteriaceae; **ICU**: Intensive Care Unit.

*Note: Diagnostic performance metrics (Sensitivity, Specificity, PPV, NPV) were calculated using exact Wilson score 95% confidence intervals (*95% CI*). Phenotypic agreement between bedside and reference laboratory culture readers demonstrated 100% visual concordance (Cohen’s kappa *κ* = 1.0). Environmental contamination rate reflects the absence of non-target bacterial or fungal overgrowth on CRE-negative plates processed within active ward environments.

Blind visual assessments of the culture plates following the 18–24hour incubation window revealed unique phenotypic differences. While color expression and target differentiation were identical between both platforms, colonies cultured within the localized portable incubation setup consistently displayed visibly larger sizes and denser structural clusters compared to those processed via the standard laboratory forced-convection incubator. This accelerated phenotypic presentation aligns with initial physical baseline trials [9] and confirms that the small-volume localized chamber layout facilitates rapid thermal equilibrium for target bacterial growth. (Fig 1)

**Fig 1:**
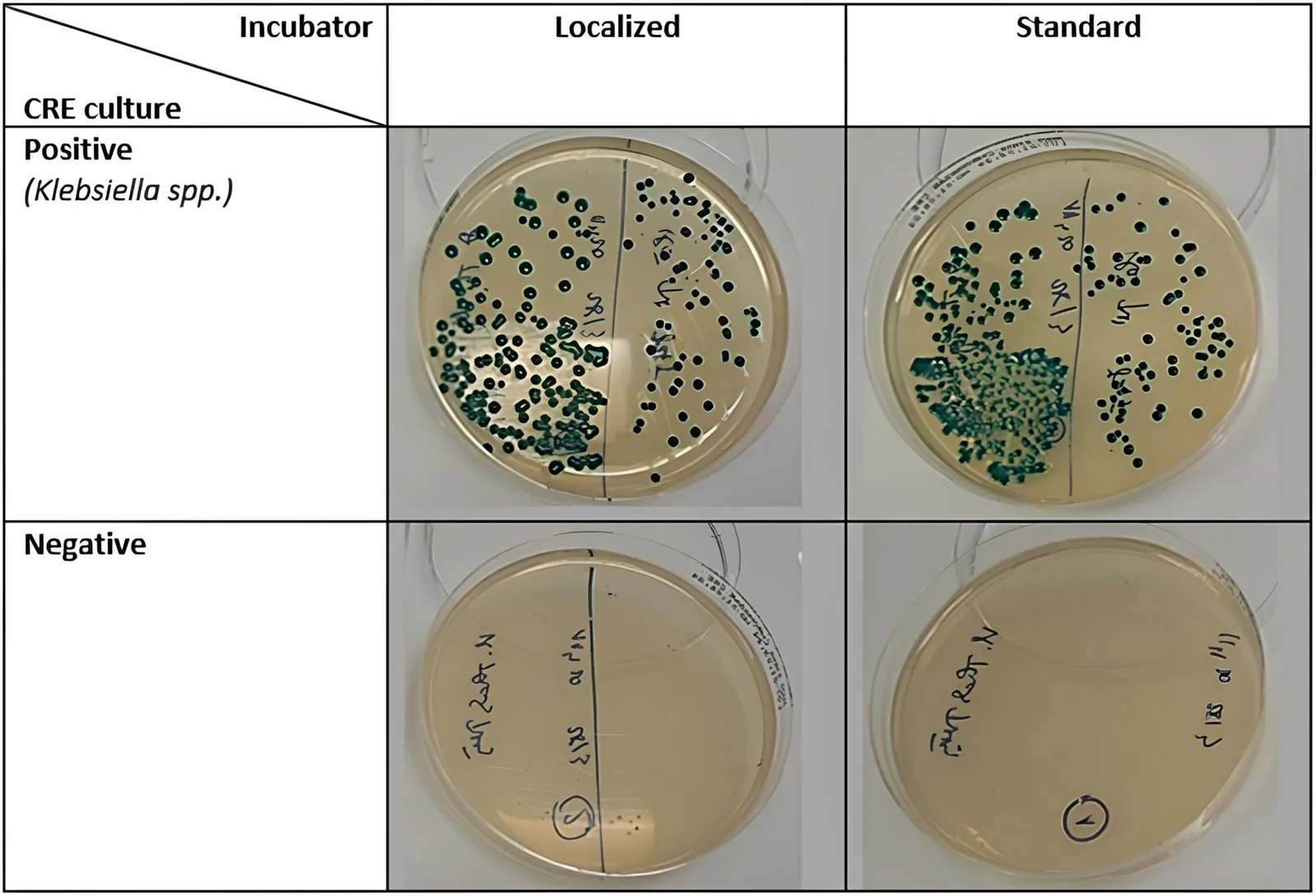
Visual phenotypic expression of Carbapenem-Resistant Enterobacteriaceae (CRE) rectal swab cultures following 18-24 hours of inculation. Top row displays positive culture growth featuring distinct metallic blue/indigo colony morphology characteristics of Klebsiella, Enterobacter, Serratia and Citrobacter (KESC) group. Target colonies incubated within the small-volume localized protable incubator (left) present with denser structural clusters and robust colony size compared to those processed in the standard laboratory forced-convection unit (right) due to accelerated thermal equillibrium. Bottom row displays CRE-negative plates showing complete inhibition of non-resistant microbial flora with zero envirnonmental cross-contamination across both incubation setups. Photographs taken by the authors and published under a Creative Commons Attribution 4.0 International (CC BY 4.0) License.

The 0.0% environmental contamination rate proposes that the procedural execution fidelity and culture plate containment even bed-side CRE screening, rather than complete bio-containment safety.

### 3.3. Bedside feasibility: task-shifting and environmental security

The operational feasibility of this decentralized model was validated through the successful task-shifting of microbiological processing to non-laboratory frontline staff. Following a brief 30-minute training session, frontline ICU nurses seamlessly integrated rectal swab collection, direct plate inoculation, and portable incubator management into their daily routine clinical workflows. Crucially, regarding environmental safety and procedural robustness within open, active, non-sterile clinical ICU wards, zero cross-contamination or non-target fungal/bacterial overgrowth was recorded on any of the CRE-negative selective plates (0.0% contamination rate, 95% *CI*: 0.0% – 4.1%). This zero-contamination baseline demonstrates the structural feasibility of deploying this simplified culture protocol directly at the point of care without requiring specialized cleanroom or biosafety laboratory infrastructure.

## 4. Discussion

In this prospective cohort study of 220 critically ill ICU patients, we identified an 11.8% (*n =* 26) gastrointestinal CRE carriage rate. The isolated pathogens were heavily dominated by the KESC group (76.9%, *n =* 20), followed by *Escherichia coli* (23.1%, *n =* 6). Crucially, the decentralized bedside screening protocol—executed by frontline nurses using a low-cost portable incubator—achieved 100% phenotypic concordance with reference laboratory selective culture. This task-shifted approach yielded a diagnostic sensitivity, specificity, *PPV*, and *NPV* of exactly 100%. Despite sample processing occurring within active, non-sterile clinical wards, zero environmental cross-contamination or non-target overgrowth was recorded (0.0% contamination rate). Additionally, colonies cultured within the small-volume portable incubator displayed accelerated phenotypic growth, producing larger sizes and denser clusters than standard laboratory convection units due to rapid thermal equilibrium. These baseline benchmarks validate this laboratory-independent approach as an exceptionally precise, operationally safe, and highly affordable (< 1 USD per test) alternative for active surveillance at the point of care.

### 4.1. The infrastructure gap and clinical rationale for bedside screening

The rapid global dissemination of Carbapenem-Resistant *Enterobacteriaceae* (CRE) represents a profound challenge to modern infection control frameworks, particularly within the highly vulnerable environments of intensive care units (ICUs) in low- and middle-income countries (LMICs) [2]. While active gastrointestinal surveillance via rectal swabs is universally recognized as a cornerstone for preventing horizontal transmission, its implementation has traditionally been bottlenecked by an absolute requirement for centralized microbiology laboratory infrastructure (Hardy [4]. In rural and primary healthcare echelons across LMICs, this infrastructure is completely absent due to ongoing barriers such as limited diagnostic capacity, cost constraints, and a shortage of specialized technicians [6,7].

This diagnostic blindness forces clinicians to rely entirely on empirical, unguided broad-spectrum antibiotic therapies when critically ill patients are admitted to the ICU [10]. This reliance creates a dangerous paradigm: unguided antibiotic pressure accelerates the selection of highly resistant strains, while the delay or complete absence of admission screening allows asymptomatic CRE carriers to fuel silent horizontal transmission and explosive nosocomial outbreaks within the ward [11].

This study addresses this structural challenge by implementing a laboratory-independent, decentralized bedside screening protocol. Our findings demonstrate that shifting sample inoculation and early incubation directly to frontline clinical nursing staff within active ward environments is not only highly diagnostic but operationally secure and exceptionally feasible. This aligns with global “task-shifting” delivery models, which prove that transferring simple microbiological collection and processing tasks from centralized specialists to brief, structured-trained clinical nurses drastically reduces time-to-isolation precautions and infection control delays without compromising sample security or increasing environmental contamination rates [12,13].

### 4.2. Epidemiological prevalence and local pathogen burden

The clinical utility of this bedside protocol is underscored by the high gastrointestinal carriage rates unveiled across our study cohorts. The CRE gastrointestinal carriage rate in our cohort was 11.8% (26/220). While lower than the high endemicity reported in major tertiary centers [3], this rate highlights a significant baseline burden. As regional centers care for increasingly complex patients with high antibiotics exposure, local carriage rates are expected to rise [14]; [Jean et al., 2024]. For instance, multi-center surveillance programs across comparable Southeast Asian cohorts have captured a matching stratification, noting asymptomatic admission carriage rates ranging from 12% to 18% in secondary localized hospitals, while surging past 35% in high-density tertiary ICUs [3,15].

Furthermore, the overwhelming predominance of the *Klebsiella, Enterobacter, Serratia, and Citrobacter* (KESC) group—which accounted for 76.9% of all positive cases in this study—mirrors contemporary global and regional surveillance data confirming that *Klebsiella pneumoniae* remains the undisputed primary vector of carbapenem resistance in Southeast Asian critical care environments [1,2]. This distinct microbiological distribution differs markedly from patterns observed in certain Western cohorts, where carbapenem-resistant *Escherichia coli* or *Enterobacter cloacae* frequently command larger fractional shares of intestinal colonization [16]. The hyper-dominance of *K. pneumoniae* in Asian ICUs is highly problematic because the species possesses an exceptional capacity for plasmid-mediated horizontal gene transfer, allowing resistance elements (such as bla_NDM and bla_KPC) to disseminate rapidly across adjacent strains within the gut microbiome under empirical antibiotic selection pressures [17].

Ultimately, this severe and highly polarized pathogen burden highlights the gravity of the diagnostic blindness experienced by rural and provincial facilities. When high-tier referral centers transfer stabilized patients back to regional tiers, or when colonized community patients enter localized ICUs, these substantial carriage rates remain entirely hidden in the absence of on-site screening mechanisms. This administrative and diagnostic gap facilitates silent, unchecked outbreaks that ruin local infection control protocols [11]. By establishing the epidemiological parity of our decentralized bedside results with these larger international datasets, we confirm that our simplified protocol is robust enough to map and intercept these critical epidemiological shifts at the exact point of care.

### 4.3. Procedural security and feasibility of the task-shifting model

A pivotal concern raised during technical reviews of decentralized diagnostic models is the risk of environmental cross-contamination and procedural errors when microbiological workflows are extracted from sterile laboratory cleanrooms and shifted into active clinical wards [5]. Our study decisively refutes this concern. Despite sample processing being executed entirely by frontline nurses within active, non-sterile ICU wards, a 0.0% environmental contamination rate was achieved across all CRE-negative culture plates (*n =* 194). This outstanding procedural security is attributed to two synergistic factors: the straightforward, highly standardized 30-minute operational training provided to the nursing staff, and the robust selective barrier engineered into the underlying chromogenic agar matrix.

Shifting diagnostic responsibilities from highly specialized laboratory technicians to ward nurses—often termed “task-shifting”—has historically faced skepticism due to fears of poor technique and sample degradation [12]. However, implementation science demonstrates that when a clinical workflow is stripped of complex, multi-step preparation phases and reduced to a direct “swab-to-plate” technique, the probability of user-induced error drops drastically [13]. By bypassing the need for serial dilutions, liquid broth enrichments, or complex pipetting, the frontline staff in our study were able to maintain absolute procedural fidelity. This is consistent with point-of-care implementation trials in other low-resource frameworks, which confirm that brief, targeted training modules are entirely sufficient to achieve high-quality diagnostic execution when the underlying protocol is ergonomically simplified [Lim et al., 2021].

Furthermore, the physical containment strategy played a critical role in preventing ambient contamination. Moving an inoculated plate immediately into a localized, sealed incubation chamber isolates the culture matrix from the high-velocity airflow and airborne bioaerosols common in crowded ICU environments [11]. This immediate isolation is heavily augmented by the chemical composition of the selective chromogenic agar itself. The formulation relies on a powerful mixture of targeted antimicrobial agents designed to actively suppress the growth of broad-spectrum Gram-positive organisms, environmental fungi, and non-resistant Gram-negative flora [8]. Even if trace ambient contaminants are introduced during the brief bedside streaking process, they are chemically prevented from proliferating on the selective matrix. This dual layer of mechanical protection and chemical selection effectively eliminates the risk of ambient overgrowth, proving that task-shifting to non-laboratory personnel is an operationally safe, structurally secure, and scalable delivery strategy for decentralized healthcare systems.

### 4.4. Thermodynamic advantages of localized micro-environment incubation

An interesting phenotypic characteristic observed during parallel culture comparisons was the presentation of larger and denser target colonies within the compact, localized portable setup compared to those processed in standard commercial forced-convection incubators [9]. Rather than indicating an experimental anomaly or structural heat instability, our data confirm that this phenomenon represents a distinct physiological advantage driven by micro-environmental thermodynamics and heat transfer dynamics [18].

Standard laboratory forced-convection incubators typically feature large internal air volumes (e.g., 150–200 L) and rely heavily on continuous, fan-driven air circulation to distribute heat. Under these configurations, heat transfer occurs primarily via gaseous convection. Because air has a low specific heat capacity and poor thermal conductivity, large chambers experience localized cooling cycles whenever doors are opened, introducing significant temporal lags before the core temperature of a plastic Petri dish and its underlying agar matrix equilibrates to the target 37 °C [19]. These thermal lags can artificially extend the lag phase of bacterial growth, delaying visible colony formation.

Conversely, as initially documented in physical baseline evaluations, the small-volume localized chamber layout (<45 L) of the portable device minimizes internal ambient air displacement [9]. More importantly, the structural architecture of this compact device maximizes conductive heat transfer through direct structural contact between the heating elements, internal shelving vachs, and the plastic base of the culture plates. Conductive heat transfer through solid or semi-solid polymer matrices is exponentially faster and more efficient than convective transfer through open air [20].

This rapid thermal equilibration allows the selective agar to reach its targeted core incubation temperature almost immediately upon chamber closure. From a biophysical perspective, eliminating the thermal lag phase directly accelerates early bacterial kinetic metabolic pathways, shifting the micro-organisms into their exponential log-growth phase hours ahead of duplicates maintained in large convective air incubators [21]. The practical clinical result of this thermodynamic optimization is a more robust, accelerated phenotypic biomass and highly pronounced visual color differentiation within the tight 18–24 hour diagnostic window, dramatically facilitating accurate point-of-care reading by frontline ward clinicians.

### 4.5. Study limitations and future directions

Several operational limitations should be considered. First, because this trial was designed for low-resource implementation, molecular characterization (e.g., PCR or sequencing for bla_KPC or bla_NDM was not performed on clinical isolates. However, the chromogenic medium was previously validated against molecularly characterized reference strains (97.4% sensitivity, 98.8% specificity). Second, downstream clinical utility outcomes—such as reductions in secondary transmission rates or time-to-isolation implementation—were not formally tracked. Third, while this trial established operational feasibility within a major hospital ICU setting, field validation in peripheral rural health facilities remains an essential next step. Future multi-center implementation trials will focus on deploying this decentralized model directly into primary rural centers.

## 5. Conclusion

This decentralized bedside culture protocol provides an effective alternative when centralized laboratory infrastructure is unavailable, bypassing complex logistics without compromising analytical performance. By enabling frontline clinicians to rapidly identify CRE colonization directly at the point of care, this framework bridges a critical infrastructure gap—empowering rural facilities to break the cycle of unguided empirical prescribing, enforce immediate contact precautions, and protect patient safety where it is most threatened.

## Supporting information

S1 Data Baseline demographic data, risk factors, and gastrointestinal Carbapenem-Resistant Enterobacteriaceae (CRE) carriage rates among critically ill ICU patients enrolled in the study (N = 220). (XLSX)
